# Linking Female Adolescents’ Knowledge, Attitudes and Use of Contraceptives to Adolescent Pregnancy in Ghana: A Baseline Data for Developing Sexuality Education Programmes

**DOI:** 10.3390/healthcare9030272

**Published:** 2021-03-03

**Authors:** Bright Opoku Ahinkorah, John Elvis Hagan, Abdul-Aziz Seidu, Thomas Hormenu, John Ekow Otoo, Eugene Budu, Thomas Schack

**Affiliations:** 1 School of Public Health, Faculty of Health, University of Technology Sydney, Sydney, NSW 2007, Australia; brightahinkorah@gmail.com; 2 Department of Health, Physical Education, and Recreation, University of Cape Coast, PMB TF0494, Cape Coast, Ghana; thormenu@ucc.edu.gh; 3 Neurocognition and Action-Biomechanics-Research Group, Faculty of Psychology and Sport Sciences, Bielefeld University, 33501 Bielefeld, Germany; thomas.schack@uni-bielefeld.de; 4 Department of Population and Health, University of Cape Coast, PMB TF0494, Cape Coast, Ghana; abdul-aziz.seidu@stu.ucc.edu.gh (A.-A.S.); budueugene@gmail.com (E.B.); 5 College of Public Health, Medical and Veterinary Sciences, James Cook University, Townsville, QLD 4811, Australia; 6 Bono Regional Health Directorate, Ghana Health Service, P. O. Box 145, Sunyani, Ghana; johnotoo.05@gmail.com

**Keywords:** adolescent pregnancy, contraceptives, Ghana, policy, predictors

## Abstract

(1) Background: Nearly one out of ten Ghanaian female adolescents aged 15–19 has experienced childbearing in urban settlements compared to twice this number in the rural populations due to unintended pregnancies. This study assessed the linkages between knowledge, attitudes, and use of contraceptives and adolescent pregnancy in one of the highly affected Municipalities (i.e., Komenda-Edina-Eguafo Abrem [KEEA]) in Ghana. (2) Methods: Employing a facility-based sampling method, 378 female adolescents aged 15–19 were selected. Unadjusted odds ratio (uOR) and adjusted odds ratio (aOR) at 95% confidence intervals (CI) and *p*-values were used for significant variables at *p* < 0.05. (3) Results: Pregnant adolescents were 2 times more likely to indicate that the procedure of procuring contraceptives is quite uncomfortable (aOR = 2.42, 95% CI = [1.29–4.55]; *p* = 0.006). Also, pregnant adolescents were 5 times more likely to have ever used traditional contraceptive methods than their non-pregnant counterparts (aOR = 5.02, 95% CI = [2.60–9.71]; *p* < 0.001). On the contrary, pregnant adolescents had lower odds of indicating that contraceptives are for only married people (aOR = 0.38, 95% CI = [0.20–0.70]; *p* = 0.002) and that it feels bad to receive contraceptive information from parents and relatives than non-pregnant adolescents (aOR = 0.42, 95% CI = [0.24–0.74]; *p* = 0.003). Pregnant adolescents were less likely to use modern contraceptives than their non-pregnant adolescents (aOR = 0.18, 95% CI = [0.11–0.31]; *p* < 0.001). (4) Conclusions: The findings indicate that female adolescents’ use of traditional contraceptives is associated with the risk of pregnancy in KEEA Municipality within the Central Region of Ghana. However, adolescents who had the perception that contraceptives are for married people and those who used modern contraceptives were less likely to get pregnant. Government and non-governmental organizations in Ghana should implement educational policies and programmes aimed at educating sexually-active female adolescents on modern contraceptives and the need to use them to prevent pregnancies. The basis for such policies and programmes should be based on evidence that compared to traditional contraceptives, modern contraceptives are more effective. In addition, there is the need to provide accurate information regarding the use of contraceptives to adolescents that will help change their attitudes towards the use of contraceptives.

## 1. Background

Adolescent pregnancy is a key risk factor to maternal and child mortality, and attributable to the vicious cycle of ill-health and poverty [1]. It is defined as pregnancy that occurs under the age of 20 years [2]. Adolescent pregnancy continues to remain a serious public health problem in both low and middle-income countries [3]. Globally, a projection of three million unsafe abortions occur yearly among adolescents aged 15 to 19 and these unsafe abortions result in considerable lasting health problems and maternal deaths [1]. Apart from complications and deaths associated with unsafe abortions, having babies during adolescence is often related to a higher risk of adverse maternal and neonatal outcomes, especially in areas where there are weak health systems [4,5]. The risks of eclampsia, puerperal endometritis, and systemic infections are higher in adolescent mothers than women aged 20 to 24 [6,7]. Adverse complications from pregnancy and childbirth are the major reasons for mortality among adolescent girls in many low and middle-income countries [1]. Stillbirths and neonatal losses are 50% higher among babies born to adolescent mothers compared to older women [8]. Infants born to adolescent mothers also have heightened chances of having low birth weight, which can have a long-term influence on their health, well-being, and development [4,9].

Globally, the adolescent birth rate has dropped from 65 births per 1000 women in 1990 to 47 births per 1000 womenin 2015 [10]. Notwithstanding this decline, there are still abortion-related complications and unintended pregnancies [1]. Each year, about 16 million girls aged 15 to 19 and 2.5 million girls below 16 years give birth and a projected 21 million girls aged 15 to 19 and 2 million girls aged under 15 become pregnant in low and middle-income countries [11,12]. The highest proportions of adolescent pregnancy in the world are recorded in sub-Saharan Africa and South-Central and Southeast Asia [1,12]. As of 2013, sub-Saharan Africa had the highest proportion of adolescent pregnancy in the world [12], attributable to the disproportionately enormous burden of sexual and reproductive health challenges that adolescent girls in sub-Saharan Africa continue to experience [13]. Births to young mothers are responsible for more than 50% of all the births, constituting 101 births per 1000 women aged 15 to19 in the region [12]. About 30% of these pregnancies occur in sub-Saharan Africa (e.g., Ghana, Nigeria, Uganda) [14,15,16,17].

According to the 2014 Ghana Demographic and Health Survey report, of the 1756 female adolescents (15 to19 years) who participated in the survey, 14% of them had ever experienced pregnancy. Out of this number, 11% had given birth while 3% were pregnant for the first time [18]. The last few years have seen no significant change in the prevalence rates reported between 2003 (14%) and 2014 (14%) respectively [18]. Regarding geographical distribution of prevalence related to adolescent pregnancy in the country, rural settlers and those living in the Brong Ahafo, Central, and Volta regions have recorded the highest figures [18]. Among the three regions with the highest rates of adolescent pregnancy, Central region has the highest rate of 7%. Within the Central Region, the KEEA Municipality is among the notable districts with high prevalence of adolescent pregnancy. Out of the total recorded pregnancies in the Municipality in 2016, 17.5% were adolescents [19]. These pregnancy patterns indicate that Ghana still records higher rates of adolescent pregnancy, especially in rural areas like the KEEA Municipality [14].

Given the importance of contraceptives in the promotion of overall health of women, including adolescents, several studies in sub-Saharan Africa have investigated the possible influences that could affect its use based on the awareness and attitudes that predict pregnancy [20,21,22,23]. Some research works have recognized sexuality knowledge, attitude, behavior, and healthcare availability and suitability as general factors for adolescents’ pregnancy [24,25]. Researchers have acknowledged family structure, cultural indulgence, familial instability, early age of marriage, inadequate knowledge of sexuality, inadequate knowledge, sociodemographic attributes, mediation skills of the persons involved, and/or the ineffectual usage of contraceptives to impact pregnancy outcome among adolescents [26,27,28,29,30]. For example, Eliason et al. [30] revealed in their study that the anxiety of side effects from contraceptives because of low awareness was identified as a primary cause of the non-use of modern contraceptives in rural Ghana. Socio-cultural norms affect attitudes (e.g., less and/or no interaction) connected to sexuality at homes and other public settings, especially among adults and young people. This situation restricts adolescents’ access to pregnancy related information surrounding sex and contraception that might lead to unintended pregnancies [31,32]. 

Despite these research attempts, there seems to be a paucity of empirical studies that have examined multiple factors concurrently from a tripartite perspective (i.e., knowledge-attitudes-use) on pregnancy risk. Generally, women who are indecisive about pregnancy have been identified to use contraceptives less incessantly and less efficiently compared to individuals who have a clearly defined and steady motivation to prevent pregnancy [33,34,35,36,37]. Hence, this study examined whether knowledge, attitudes, and use of contraceptives predict adolescent pregnancy in the KEEA Municipality of Ghana. Findings from this study would provide some baseline information to develop pragmatic community-based interventions to decrease adolescent pregnancies through empirically driven initiatives within KEEA Municipality and perhaps, other homogenous settlements elsewhere in the country.

## 2. Materials and Methods 

### 2.1. Study Design 

A case-control research design with a ratio of 1:1 matching was employed for this study. Any female adolescent aged between 15 to19 who was identified to be pregnant during the study period and attended antenatal clinic in the KEEA Municipality was referred to as a case in this study. Comparatively, a female adolescent counterpart within the same age (15 to 19) within the same study area who has never been pregnant was considered as a control. To ensure homogeneity between groups, respondents were asked of their marital status, age, and sexual experience and they gave verbal responses to these questions. The three main inclusion criteria: not to be married (because in the Ghanaian social context, pregnancy is acceptable within the context of marriage), to have had sex and be aged 15 to 19. The control group respect to the age and marital status criteria but has never been pregnant. The two groups were identified retrospectively and aimed to determine risk factors accounting for adolescent pregnancy [38]. It was shown that high knowledge, positive attitude, and the use of contraceptives were protective against pregnancy [33,34,35]. The Research and Ethics Committee of University of Cape Coast, Ghana (UCCIRB/CES/2016/04) and the Ghana Health Service Ethics Review Committee gave ethics approval for this study (GHS-ERC: 13 December 2016). Written and verbal informed consent was obtained from all participants.

### 2.2. Study Area

The study was carried out in the KEEA Municipality of Ghana. Statistics from 2010 Population and Housing Census indicate that the population of KEEA Municipality was 144,705, forming 6.6% of the Central Region’s overall population. Males represent 48.2% of the entire population compared to females whose numbers constitute 51.8%. Sixty four percent (64%) of the sample is considered rural. The Municipality has a youthful population with about 40% of the populace below 15 years, indicating a general population pyramid which dwindles with a lesser number of ageing persons (8.6%). The Municipality has General Fertility Rate and Total Fertility Rate of 105.0 births per 1000 females aged 15–49 years and 3.6, respectively. The KEEA Municipality’s Crude Birth Rate (CBR) is also 24.6 per 1000. 

The Municipal Health Management Team (MHMT) coordinates the public health services at the Municipality, providing support to sub-district on disease prevention and management, health promotion, and general public health education. Most of the health facilities in the Municipality are situated in Elmina, the Municipal capital. Geographical accessibility to healthcare is a foremost challenge within the municipality due to the relatively large geographical area [39]. 

### 2.3. Population and Sampling 

The study population was composed of 15 to 19-year-old female adolescents in the KEEA Municipality, both pregnant and non-pregnant. Out of the population of 7667, the Krejcie and Morgan [40] table for determining sample sizes was used to obtain a sample size of 400. The participants were selected from the respondents of a questionnaire. From the 400 enquiries submitted, information was retrieved from 378 respondents, representing 189 pregnant and 189 non-pregnant adolescents. The response rate was 95%. A facility-based sampling approach was used across five health facilities. The sampling method used herein was defined as a process of drafting members of a target population from several health facilities, including correctional and drug treatment centers, sexually transmitted diseases clinics or general health centers, and hospitals [41]. 

Within the framework of this sampling procedure, pregnant adolescents undergoing antenatal services from the five selected health facilities were initially chosen purposively. For each pregnant adolescent chosen, a non-pregnant adolescent counterpart within the same age group (15 to 19), accessing other healthcare services apart from antenatal care services, was suitably identified and drafted from the identified five health facilities. The health facilities from where the sampling was done were Kissi health centre, Komenda health centre, Elmina urban health centre, Agona health centre, and Ankaful General Hospital. From each health center, an equal number of females, both pregnant and non-pregnant, was chosen in a total of 400 adolescents, although 22 withdrew from the data collection exercise due to personal reasons.

### 2.4. Instrumentation

A questionnaire was used to collect data for this study. The items on the questionnaire were obtained from empirical information gathered from previous studies [33,34,35,42,43,44,45,46]. The questionnaire consisted of three main sections. Section A looked at the knowledge of respondents on contraceptives. Section B focused on the attitudes toward contraceptive use. In section C, questions aimed to ascertain the use of modern contraceptive methods (e.g., female sterilization, male sterilization, IUD, injectables, implants, pill, condom, diaphragm, foam/jelly, and other modern methods) or traditional (i.e., rhythm method, withdrawal, and other traditional methods) of contraception. To ensure both the content and construct validity of the questionnaire, a thorough literature review on how knowledge, attitudes and use of contraceptives are related to adolescent pregnancy was done before the development of the questionnaire to ascertain the association between these factors and adolescent pregnancy. Potential factors were identified using the information obtained from literature. Also, the questionnaire was given to three national experts in adolescent health education and promotion, for their inputs to guarantee that the individual item structure and related content of the instrument reflected the specific objectives of the study [47]. Before being applied, the questionnaire was pre-tested among 50 female adolescents in the Cape Coast Metropolis with homogenous characteristics as the main study population. Some of the questions deemed unclear were modified to suit the study context. The Kuder–Richardson formula 21 (KR-21) was then used to generate the instrument’s internal reliability coefficient, 0.75, a suitable value [48].

### 2.5. Procedure

The data collection procedure was scheduled with the medical administrators of the five identified health facilities, to ensure that their daily operations (i.e., health delivery services) were not interrupted. Two of the researchers and two research assistants, trained for 3 days, carried out the data collection exercise consistently for six weeks. The purpose of the study was clearly explained to each participant. After giving every pregnant adolescent a questionnaire, a non-pregnant “counterpart” or “match” was selected to fill a questionnaire at the same center. The filling of the questionnaire was done in unison in a seminar room of the health center. The data collection team gave standard instructions about the questionnaire to all participants, who gave informed consent for the participation. The respondents were informed that participation was voluntary, and they could withdraw from the study at any time they felt unsecured. The trained assistants, proficient in both English and the local dialect, facilitated the administration of the questionnaire to some study participants, especially those who were not proficient in English and might require help with the interpretation of some contents in their native language. Anonymity, respect and confidentiality, as part of ethical considerations in the study, were fully preserved throughout the data collection process by removing any peculiar identifier from the research instrument.

### 2.6. Data Analysis

All completed questionnaires were thoroughly cleaned for accuracy by the researchers. Each filled questionnaire was then coded on a pre-arranged data coding sheet by the researchers to reduce biases. Data were subsequently imputed into SPSS version 21 (SPSS, Chicago, IL, USA) for analysis. Chi-square test of independence and binary logistic regression were used, since the outcome variable was binary (Pregnant or Not-pregnant). Response categorization to all the independent variables was also a binary scale (e.g., Yes or No; Agree or Disagreet). The outcome of interest was pregnancy. Hence, the interpretation of results of the binary logistic regression was in reference to the pregnant adolescents. Chi-square was initially carried out between the dependent variable (i.e., pregnancy status) and independent variables (i.e., knowledge, attitudes and use of contraceptives) to identify their relationship. The independent variables that were statistically significant with the dependent variable were further entered in a binary logistic model to ascertain their degree of effecton the dependent variable. For comparison purposes, both unadjusted odds ratio (uOR) and adjusted odds ratio (aOR) at 95% confidence intervals (CI) and *p*-values were reported and pegged for significant variables at *p* < 0.05. The binary logistic regression was employed to find out how the variables that measure knowledge, attitudes and use of contraceptives in this study are connected to adolescent pregnancy as well as indicate the degree and direction of the association between the variables.

## 3. Results

### 3.1. Distribution of Adolescent Pregnancy across Knowledge, Attitudes, and Use of Contraceptives

Table 1 shows the results of the association between knowledge, attitudes and use of contraceptives and adolescent pregnancy in the KEEA Municipality. The results show that most of the pregnant adolescents had knowledge about traditional contraceptive methods (61.2%) compared to the non-pregnant adolescents (38.8%; *p* < 0.001). Again, 69.0% of the non-pregnant adolescents agreed that contraceptives are for only adult married people, compared to pregnant adolescents (31.0%; *p* < 0.001). The perception that adolescents who use contraceptives are bad was high among non-pregnant adolescents (64.4%) compared to the pregnant adolescents (35.6%; *p* < 0.001). A majority of the non-pregnant adolescents agreed that it feels bad to receive contraceptive information from parents and relatives (70.2%), compared to pregnant adolescents (29.8%; *p* < 0.001). Approximately 54% of the pregnant adolescents were of the view that the procedure of procuring contraceptives is often uncomfortable while 46.4% of the non-pregnant adolescents had that view (*p* = 0.003). The use of modern contraceptives was high among the non-pregnant adolescents (67.7%), compared to the pregnant adolescents (32.3%; *p* < 0.001). Only 20% of the non-pregnant adolescents indicated to have used traditional contraceptive methods, while 80% of the pregnant adolescents were affirmative on that (*p* < 0.001). From the Chi-square test results, all the independent variables showed significant association with adolescent pregnancy (see Table 1), except the knowledge about modern contraceptive methods, and the perception that contraceptive use leads to infertility.

### 3.2. Binary Logistic Regression on Knowledge, Attitudes and Use of Contraceptives on Adolescent Pregnancy

Table 2 contains the results of an unadjusted binary logistic regression analysis on the association of knowledge, attitudes and use of contraceptives on adolescent pregnancy in KEEA Municipality. All the independent variables that were included in the binary logistic regression analysis revealed statistically significant associations with adolescent pregnancy. Pregnant adolescents were more likely to have knowledge of traditional contraceptive methods than non-pregnant adolescents (uOR = 2.87, 95% CI = [1.88–4.36]; *p* < 0.001). Pregnant adolescents were two times more likely to state that the process of acquiring contraceptives is often embarrassing (uOR = 2.15, 95% CI = [1.30–3.56]; *p* = 0.003). Also, pregnant adolescents were 7 times more likely to have ever used traditional contraceptive methods compared to non-pregnant adolescents (uOR = 6.60, 95% CI = [3.86–11.30]; *p* < 0.001). On the contrary, pregnant adolescents had lower odds of perceiving that “adolescents who use contraceptives are bad” than non-pregnant adolescents (uOR = 0.29, 95% CI = [0.19–0.41]; *p* < 0.001). Further, pregnant adolescents were less likely to mention that it feels bad to receive contraceptive information from parents and relatives than non-pregnant adolescents (uOR = 0.28, 95% CI = [0.18–0.45]; *p* < 0.001). Pregnant adolescents were less likely to use modern contraceptives compared to non-pregnant adolescents (uOR = 0.24, 95% CI = [0.16–0.37]; *p* < 0.001).

Table 3 contains the results of an adjusted binary logistic regression analysis on the association of knowledge, attitudes, and use of contraceptives on adolescent pregnancy in KEEA Municipality. The results indicate that pregnant adolescents were 2 times more likely to indicate that the process of acquiring contraceptives is often embarrassing (aOR = 2.42, 95% CI = [1.29–4.55]; *p* = 0.006). Additionally, pregnant adolescents were 5 times more likely to have ever used traditional contraceptive methods compared to non-pregnant adolescents (aOR = 5.02, 95% CI = [2.60–9.71]; *p* < 0.001). On the contrary, pregnant adolescents had lower odds of indicating that contraceptives are for only married people (aOR = 0.38, 95% CI = [0.20–0.70]; *p* = 0.002) and it feels bad to receive contraceptive information from parents and relatives than non-pregnant adolescents (aOR = 0.42, 95% CI = [0.24–0.74]; *p* = 0.003). Pregnant adolescents were less likely to use modern contraceptives compared to non-pregnant adolescents (aOR = 0.18, 95% CI = [0.11–0.31]; *p* < 0.001).

## 4. Discussion

This study assessed the association between knowledge, attitudes and use of contraceptives and adolescent pregnancy in the KEEA Municipality of Ghana. The findings of the study show that knowledge and use of traditional contraceptive methods, attitudes toward the use of contraceptives are associated with adolescent pregnancy in the KEEA Municipality of Ghana.

Specifically, findings suggest that 48.7% of the pregnant adolescents had knowledge on modern contraceptive methods whereas 61.2% had knowledge on traditional contraceptive methods. In terms of use of contraceptives, while 32.3% of the pregnant adolescents had ever used modern contraceptives, 80% had ever used traditional contraceptives. The knowledge and use of traditional method of contraception were found as risk factors for adolescent pregnancy and the findings have been confirmed in previous studies (e.g., [28,49,50]). Several studies e.g., [28,51,52,53] showed that even though adolescents are usually knowledgeable on different contraceptives approaches, the choice of the contraceptive method is mostly dependent on how well they are informed. For example, research on sexual behaviour and contraceptive usage among female adolescents aged 14–21 years in Nigeria revealed that these girls preferred coitus interruptus (withdrawal) and the rhythm method to other methods. Similarly, Ajayi et al. [54] reported that many women inNigeria choose ineffective traditional contraceptive procedures over more effective modern contraceptive techniques which was attributed to a possible fear of the negative side effects associated with modern contraceptive methods, a decreased sensitivity associated with the use of condoms or parents’ disapproval of the use of artificial contraception [55,56]. 

On the contrary, we found that pregnant adolescents were less likely to use modern contraceptives, compared to non-pregnant adolescents, which supports the findings of previous studies [57,58]. Modern methods of contraception have been shown to be efficient means of avoiding unplanned pregnancies, compared to traditional methods. This is against the general knowledge that the effectiveness of the rhythm method could be compromised by the use of medications like anxiolytics, antidepressants, nonsteroidal anti-inflammatory drugs (NSAIDS), or certain antibiotics. These drugs are likely to affect the timing of ovulation [7,58]. Again, coitus interruptus has been considered as one of the least efficient methods since correct timing of withdrawal is usually problematic to regulate, responsible for the possibility of ejaculating while inside the vagina [7]. Current findings underscore the need to educate sexually active adolescent girls on modern contraceptives at both community and school levels as well as provide user friendly services and/or mechanisms that would encourage them to use these modern methods. Knowing the implications of traditional methods of contraception, their usage could be discouraged.

Pregnant adolescents were more likely to have positive attitudes toward the use of contraceptives. Specifically, pregnant adolescents were less likely to have the perception that contraceptives are for only adult married persons, adolescents who use contraceptives are bad and it feels bad to receive contraceptive information from parents and relatives. Supported by previous studies (e.g., [59]), the adolescents with positive attitudes toward contraception may have strong desire to use contraceptives and hence, are less likely to become pregnant. However, the fact that pregnant adolescents show positive attitudes toward the use of contraceptives suggests that their positive attitudes do not necessarily lead or translate to its use, or even if they do, pregnant adolescents could be using ineffective contraceptive methods [26,28,51,53]. Despite these findings, it is important to mention that there are measures that have been used globally such as the variations of the London Measure of Unplanned Pregnancy (LMUP) [60] to measure pregnancy intention. We therefore recommend the use of such measures to examine the pregnancy intentions of adolescents in the KEEA municipality.

Pregnant adolescents compared to non-pregnant adolescents were more likely to agree to the notion that the process of acquiring contraceptives is often embarrassing. This may explain the perception of a barrier towards the access to contraceptives and adolescent pregnancy in most developing countries, including Ghana [61,62,63]. Hence, even when there is easy access to contraceptives, uptake may be inhibited by societal stigma associated with non-marital sexual activity, the burden to demonstrate fertility and an overall lack of agency to make rightful choices. Such barriers explain the high unmet need for contraception among sexually active adolescent girls in Ghana [64,65], which according to Nyarko [66], could account for the high unintended pregnancy in Ghana. The available national statistics show that, each year, approximately 750,000 teenagers between the ages of 15 and 19 years-old get pregnant [18,67]. Recent empirical evidence suggests that the KEEA Municipality is one of the districts well-known for the high prevalence of adolescent pregnancy in Ghana [14,68]. Therefore, removing the barriers toward access to contraceptives is critical in reducing adolescent pregnancy in the KEEA Municipality.

### 4.1. Limitations

The present study has some methodological issues that may have influenced our results. First, female adolescents might possibly not give honest responses to questions due to the delicate nature of the research topic [69,70]. This could be more evident among the non-pregnant adolescents whose pregnancy status was obtained through verbal responses without any clinical verification. However, the procedure used for data collection (using detached seats, anonymous data collection) could get the most out of getting truthful answers from the respondents. Second, due to the contraceptive method combinations (e.g., modern versus traditional methods) used in this study, the distinctiveness of interchanging and/or concurrently using these methods may not have been reported by all study participants. Hence, the results might be liable to response error and recall bias [14,50]. Third, by using a case-control design, causal associations could not be drawn between study variables. For example, determining whether female adolescents’ attitudes and experiences preceding the usage of a method choice or their current unpredictable use of a method could not be ascertained. However, reported attitudes may mirror (serve as a proxy) for attitudes and experiences that were pertinent at the time of noting a current method or otherwise [33]. These weaknesses were reduced by selecting cases and controls that had similar characteristics beside pregnancy. Additionally, the study was carried out in five health facilities in the KEEA Municipality, hence it will be inappropriate to generalize the findings to other settings [14]. We also acknowledge that there are other adolescents who are in the community who did not attend health facilities. Notwithstanding, the health facilities where data collection took place were located within the major communities within the municipality and hence the adolescents sampled from the health facilities to a large extent lived within the communities and have characteristics that may not differ considerably from adolescents who were not at the health facilities at the time of the data collection. Therefore, the results from the sampled adolescents can still be relevant for generalization to all adolescents in the communities. Based on the focus of the study, important socio-economic and anamnestic characteristics of the study participants were not gathered. This may affect the findings.

### 4.2. Practical Implications

Current findings underscore the significance of adequate knowledge of modern contraceptives, positive attitudes towards the use of contraceptives and modern contraceptive use in the prevention of adolescent pregnancy. Hence, interventions aimed at reducing adolescent pregnancy may be dependent not on contraceptives in general, but on method type and consistent compliance to the method’s requirements and a correct usage [71]. Significant factors that may account for the lack of contraceptive or inconsistent use include lack of information and misconceptions about the use or suitability of contraception. There is the need for strengthening the establishment of family planning information and services for adolescent girls in the KEEA Municipality. For instance, in many typical Ghanaian homes, interactions on matters such as sex and related issues are forbidden with outright overt societal disapproval [26,28]. Hence, pregnancy related interventions should prioritize attitudes toward contraception, especially attitude towards modern methods and encourage the use of modern contraceptives.

## 5. Conclusions

This study shows that female adolescents’ knowledge and use of traditional contraceptives are linked to the risk of pregnancy in KEEA Municipality of the Central Region of Ghana. However, adolescents who had the perception that contraceptives are for married people and those who used modern contraceptives were less likely to get pregnant. Government and non-governmental organizations in Ghana should implement educational policies and programmes aimed at educating female adolescents on modern contraceptives and the need to use them to prevent pregnancies. The basis for such policies and programmes should be based on evidence that compared to traditional contraceptives, modern contraceptives are more effective. In addition, there is the need provide accurate information regarding the use of contraceptives to adolescents that will help change their attitudes towards the use of contraceptives. Future research could use longitudinal and qualitative research designs to adequately unearth indebt sociocultural antecedents and behaviors related to contraceptive use and pregnancy risk.

## Figures and Tables

**Table 1:** Distribution of Adolescent Pregnancy across Knowledge, Attitudes, and Use of Contraceptives.

Variables	Pregnant N (189) %	Non-Pregnant N (189) %	*χ^2^*	*p*-Value
Knowledge about modern contraceptive methods				
No	60.0	40.0	1.187	0.276
Yes	48.7	51.3		
Knowledge of traditional contraceptive methods				
No	35.5	64.5	24.767	<0.001
Yes	61.2	38.8		
Contraceptives are for only adult married persons				
Disagree	62.7	37.3	37.001	<0.001
Agree	31.0	69.0		
Adolescents who use contraceptives are bad				
Disagree	65.3	34.7	33.183	<0.001
Agree	35.6	64.4		
Contraceptive use leads to infertility				
Disagree	50.8	49.2	0.134	0.714
Agree	48.8	51.2		
It feels bad to receive contraceptive information from parents and relatives				
Disagree	59.9	40.1	31.125	<0.001
Agree	29.8	70.2		
The process of acquiring contraceptives is often embarrassing				
Disagree	34.9	65.1	8.984	0.003
Agree	53.6	46.4		
Ever used modern contraceptive methods				
No	33.9	66.1	43.404	<0.001
Yes	32.3	67.7		
Ever used traditional contraceptive methods				
No	37.7	62.3	54.207	<0.001
Yes	80.0	20.0		

**Table 2:** Unadjusted Association between Adolescents’ Knowledge, Attitudes and Use of Contraceptives on pregnancy.

Variables	B	Wald	uOR (CI)	*p*-Value
Knowledge of traditional contraceptive methods				
No	1.05	24.19	Ref	
Yes			2.87 (1.88–4.36)	<0.001
Contraceptives are for only adult married persons				
Disagree	−1.32	35.55	Ref	
Agree			0.27 (0.17– 0.41)	<0.001
Adolescents who use contraceptives are bad				
Disagree	−1.23	32.15	Ref	
Agree			0.29 (0.19–0.45)	<0.001
It feels bad to receive contraceptive information from parents and relatives				
Disagree	−1.26	29.76	Ref	
Agree			0.28 (0.18–0.45)	<0.001
The process of acquiring contraceptives is often embarrassing				
Disagree	0.76	8.77	Ref	
Agree			2.15 (1.30–3.56)	0.003
Ever used modern contraceptive methods				
No	−1.41	41.64	0.24 (0.16–0.37)	<0.001
Yes				
Ever used traditional contraceptive methods				
No	1.89	47.42	Ref	
Yes			6.60 (3.86–11.30)	<0.001

uOR = unadjusted Odds Ratio; CI = Confidence Interval; Ref = Reference Category.

**Table 3:** Adjusted Association between Adolescents’ Knowledge, Attitudes, and Use of Contraceptives on pregnancy.

Variables	B	Wald	aOR (CI)	*p*-Value
Knowledge of traditional contraceptive methods				
No	0.43	2.37	Ref	
Yes			1.54 (0.89–2.65)	0.124
Contraceptives are for only adult married persons				
Disagree	−980	9.57	Ref	
Agree			0.38 (0.20–0.70)	0.002
Adolescents who use contraceptives are bad				
Disagree	−578	3.46	Ref	
Agree			0.56 (0.31–1.03)	0.063
It feels bad to receive contraceptive information from parents and relatives				
Disagree	−862	9.10	Ref	
Agree			0.42 (0.24–0.74)	0.003
The process of acquiring contraceptives is often embarrassing				
Disagree	0.89	7.58	Ref	
Agree			2.42 (1.29–4.55)	0.006
Ever used modern contraceptive methods				
No	−1.72	39.59	0.18 (0.11–0.31)	<0.001
Yes				
Ever used traditional contraceptive methods				
No	1.61	23.06	Ref	
Yes			5.02 (2.60–9.71)	<0.001

aOR = adjusted Odds Ratio; CI = Confidence Interval; Ref = Reference Category.

## Data Availability

According to the Institutional Review Board, University of Cape Coast, Ghana and ethical guidelines of the Ghana Health Service, public data sharing, even anonymous, is sometimes restricted by participants’ written informed consent.

## Abbreviations

KEEA: Komenda-Edina-Eguafo-Abrem; OR: Odds Ratio; CI: Confidence Interval; MHMT: Municipal Health Management Team; CBR: Crude Birth Rate.
